# Multidimensional economic deprivation during the coronavirus pandemic: Early evidence from the United States

**DOI:** 10.1371/journal.pone.0244130

**Published:** 2020-12-16

**Authors:** Shatakshee Dhongde

**Affiliations:** School of Economics, Georgia Institute of Technology, Atlanta, GA, United States of America; Universidade Federal de Minas Gerais, BRAZIL

## Abstract

The coronavirus pandemic led to a severe economic shock in the United States. This paper uses a unique survey data collected early on in the pandemic to measure economic deprivation among individuals. The Federal Reserve Board fielded a Survey of Household Economics and Decision-making (SHED) in April 2020. This survey is used to compile data on four indicators of economic deprivation, namely: i) Overall financial condition, ii) Loss of employment, iii) Reduction in income and iv) Inability to pay bills in full. Data on these indicators is compiled for each individual and is used in a novel way to construct a set of multidimensional deprivation indices. These indices measure the overlap of deprivations experienced by an individual. Results show that almost 25 percent of the respondents faced hardships in at least two of the four indicators. More than 13 percent of adults reported their inability to pay monthly bills and struggled to make ends meet financially. One in four respondents had lower income compared to income from previous month. The economic shock affected Hispanics in a more profound way. More than 37 percent Hispanics reported hardship in two or more indicators and 8 percent reported hardship in all four indicators. Higher proportion of young adults and those without a college degree suffered multiple hardships. The paper highlights the plight of Americans during the early months of the economic crisis set in motion amid the coronavirus pandemic and sheds light on how economic disparities deepened along racial/ethnic lines.

## I. Introduction

Between January 1, 2020 and December 1, 2020, more than 13 million cases of coronavirus disease 2019 (Covid-19) were reported in the United States by the Center for Disease Control (CDC) [1]. Even more sobering news is that during this period, more than 266,000 died from Covid-19 in the United States. So far, the United States has been the hardest hit country by the pandemic. At the peak of the virus, the death rate in the United States exceeded 2000 per day. The pandemic triggered an unprecedented economic crisis in the country. As businesses, large and small, were forced to shut down, many Americans lost their jobs, struggled to pay rents and had to rely on community food banks for basic needs. The national unemployment rate rose by 10.3 percentage points over the month of April 2020 to 14.7 percent [2].

This paper uses a unique data, the Survey of Household Economics and Decision-making (SHED), collected by the Federal Reserve Board [3]. The survey was fielded in April 2020, to gauge the changes in financial conditions of households since the start of the coronavirus pandemic. The SHED dataset is used to compile data on four indicators of economic deprivation, namely: i) Overall financial condition, ii) Loss of employment, iii) Reduction in income and iv) Inability to pay bills in full. Based on these four indicators, multidimensional measures of economic deprivation are estimated. A multidimensional index measures the extent of deprivation experienced by an individual in multiple indicators simultaneously. Previously, multidimensional indices have been used in the United States to measure changes in poverty over time [4–7]. This paper is the first to use a multidimensional framework in a novel way; it uses qualitative data from survey responses to measure economic deprivation in the midst of the coronavirus pandemic in the United States.

The SHED survey was conducted early on in the pandemic (between April 3 and April 6, 2020). CDC data shows that as of April 6, 2020, around 374,329 positive cases and 12,064 deaths were confirmed in the United States. The Johns Hopkins Coronavirus Research Center [8] also shows similar numbers; about 367,200 cases and 14,200 deaths as of April 6, 2020. In addition to the spread of the pandemic, individuals’ wellbeing was also severely affected by the resulting economic slowdown. The rising number of infections and deaths prompted several states to issue lock down orders. In the United States, stay-at-home orders were issued in just nine states by March 23, 2020. Within a week, by March 30, 2020, 30 states had issued these orders. By April 6, 2020, at least 316 million people in at least 40 states, the District of Columbia and Puerto Rico were being urged to stay home [9]. As the economic activity came to a near standstill, economic hardships increased rapidly. The timing of the SHED survey is also important because the Coronavirus Aid, Relief, and Economic Security (CARES) Act, which was passed on March 27, 2020, was still in the process of implementation and most benefits were not yet received. Thus, the survey provides a snapshot of individuals nationwide who were facing economic hardships.

Our results indicate that from March 2020 through early April 2020, 12 percent of adults reported losing a job or being furloughed. More than 20 percent individuals said that they were just getting by or finding it difficult to get by financially. Financial stress was evident from other indicators such as lower incomes compared to previous month (23 percent) and the inability to pay bills in full (almost 15 percent). The multidimensional index shows that 25 percent of the respondents were struggling in two or more indicators of economic hardship. Young adults and those without a college education in particular, experienced a disproportionate loss of economic wellbeing.

The paper contributes to the growing literature on the economic fallout of the pandemic (see [10] for an extensive review). In the United States, more than a hundred studies have been listed on the National Bureau of Economics Research working papers series [11]. Carlsson-Szlezak et al. [12] suggested three ways in which the pandemic affected countries’ economies. The first impact was due to reduction in consumption of goods and services because of social distancing measures and overall lower consumer confidence during the pandemic. The second effect was through financial market shocks and their effects on the real economy. A third effect was due to supply-side disruptions in production of goods and services due to COVID-19. There is increasing evidence, which shows that consumer spending in the United States sharply reduced in sectors such as hotels, transportation and food-services, which require in-person interaction (e.g. [13–15]). Other sectors, which did not require physical contact–such as landscaping, construction or financial services, experienced much smaller losses. In Italy, mobility trends associated with tourism, retail, and services experienced a sudden contraction of more than 90% during the lockdown. Bonacorssi et al. [16] found that in Italy, the impact of lockdown measures was stronger in municipalities where inequality was higher and income per capita was lower. Coibion et al. [17] predicted a severe rise in the unemployment rates and a severe fall in the labor participation rate. Martin et al. [18] used a micro-economic model and predicted that in the absence of social protection, poverty rates in the San Francisco Bay Area would increase significantly. However, the impact of the lockdown was not limited to lower income households. Cox et al. [19] found that during the initial stages of the pandemic in March 2020 there were large declines in spending among consumers across all quartiles of the income distribution. Estimates of the multidimensional deprivation index in this paper reveal that even among individuals with incomes in excess of $100,000, 31 percent adults were deprived in at least one indicator, and 13 percent were deprived in two or more indicators.

The paper underscores the fact that early on in the crisis, individuals belonging to different race/ethnicity suffered financial setbacks to varied extent. Unemployment rates were particularly high among Hispanics. Nearly one-third of the Hispanics reported that overall they were just getting by or finding it difficult to get by financially. Emerging data from cities such as Chicago, Detroit and New York City shows that the coronavirus is affecting Blacks and Latinos in much greater proportions [20]. A CDC Report [21] used county level data between February-June 2020 and found a disproportionate number of Covid-19 cases among underrepresented racial/ethnic groups. Hispanics were the largest population group followed by Blacks living in hotspot counties with a disproportionate number of cases. Consistent with this evidence, the paper finds that economic deprivation was more pronounced among Black and Hispanic respondents. More than 20 percent of Hispanic respondents and 17 percent of Black respondents were in a precarious financial condition and were struggling to pay bills in full in the early months of the pandemic. However, once factors such as household income, size, and individual’s age, education, are controlled for, the regression estimates show that race or ethnicity are no longer statistically significant in predicting the number of deprivations experienced by individuals. This suggests that low income and education levels among minority population made them even more vulnerable to the economic hardship resulting from the pandemic.

The remainder of the paper is structured as follows. Section 2 provides details about the SHED survey and summarizes the four indicators of economic deprivation used in the analysis. Multidimensional indices used in the analysis are explained and illustrated in Section 3. Economic deprivation in the entire sample is discussed in Section 4, and deprivation by race and ethnicity is discussed in Section 5. The overlap between deprivations in different indicators is analyzed in Section 6. Regression analysis on factors related to the number of deprivations is summarized in Section 7. Section 8 concludes.

## II. Data

The Federal Reserve Board has conducted SHED survey each year in fall since 2013. In addition to the full survey, the board conducted a supplemental survey over the first weekend in April, in the midst of the coronavirus pandemic. Compared to the full survey, which typically interviews more than 12,000 adults (aged 18 and above), the supplemental survey in April 2020 recorded responses from a smaller sample consisting 1,030 adults. Survey respondents were adults aged 18 and above. The supplemental survey was fielded from April 3 through April 6, 2020. Of the 2,853 panel members contacted to take the survey, 1,030 participated, yielding a final-stage completion rate of 36.1 percent [3]. The final stage completion rate is lower than it was for the full survey because the supplemental survey was conducted over a single weekend in order to obtain timely results. After the survey collection was complete, weights in a post-stratification process were adjusted in order to correct for any survey non-response as well as any non-coverage or under- and over-sampling in the study design.

The supplemental survey was shorter in length than the full survey and was comprised of 13 questions. Questions in the supplemental survey were specifically aimed at understanding how the pandemic affected families’ economic conditions. Not all questions in the supplemental survey are exactly comparable with those in the full survey. Thus, it is difficult to compare the results of this survey with the results of the full survey. The same sampling and weighting approaches were applied to the supplemental survey as have been applied for the main survey. Weights provided in the survey for each individual observation are used in the analysis. These weights allow the entire sample to reflect the observable characteristics of the adult population in the United States.

The paper compiles data on questions from the supplemental survey of April 2020, which broadly reflect different aspects of economic deprivation. Table 1 lists these questions and the choice of responses for each of the questions. Questions and responses in Table 1 are exactly replicated from the survey questionnaire. Each individual in the survey choses just one response from the available options. If an individual has chosen a response highlighted in bold, then he/she is considered deprived in that particular indicator. Overall, weak financial condition is signaled when an individual is a) just getting by or b) finding it difficult to get by. Loss of employment in the last week is identified as a) temporarily laid off or furloughed or b) not employed but looking for a job. Another indicator of financial stress is when an individual’s income is a) somewhat lower or b) much lower than income compared to last month’s income. The fourth indicator is an individual’s inability to pay monthly bills in full.

**Table 1:** Indicators chosen to measure economic deprivation.

Indicators	Survey Questions	Responses
Financial Condition	Overall, which one of the following best describes how well you are managing financially these days?	1. Living comfortably
		2. Doing okay
		**3. Just getting by**
		**4. Finding it difficult to get by**
Employment Status	Which one of the following best describes your employment status last week?	1. Employed
		2. Self-employed
		3. Not working, but being paid my normal wages
		**4. Temporarily laid off or furloughed**
		**5. Not employed, but looking for a job**
		6. Not employed and not looking for a job
Change in Income	How did your income last month (March) compare to your income two months ago (February)?	1. Much higher
		2. Somewhat higher
		3. About the same
		**4. Somewhat lower**
		**5. Much lower**
Ability to Pay Bills	Which best describes your ability to pay all of your bills in full this month?	1. Able to pay all bills
		**2. Cannot pay some bills**

Source: Federal Reserve Board Report [3]. Responses in bold indicate deprivation in that indicator.

**Fig 1:**
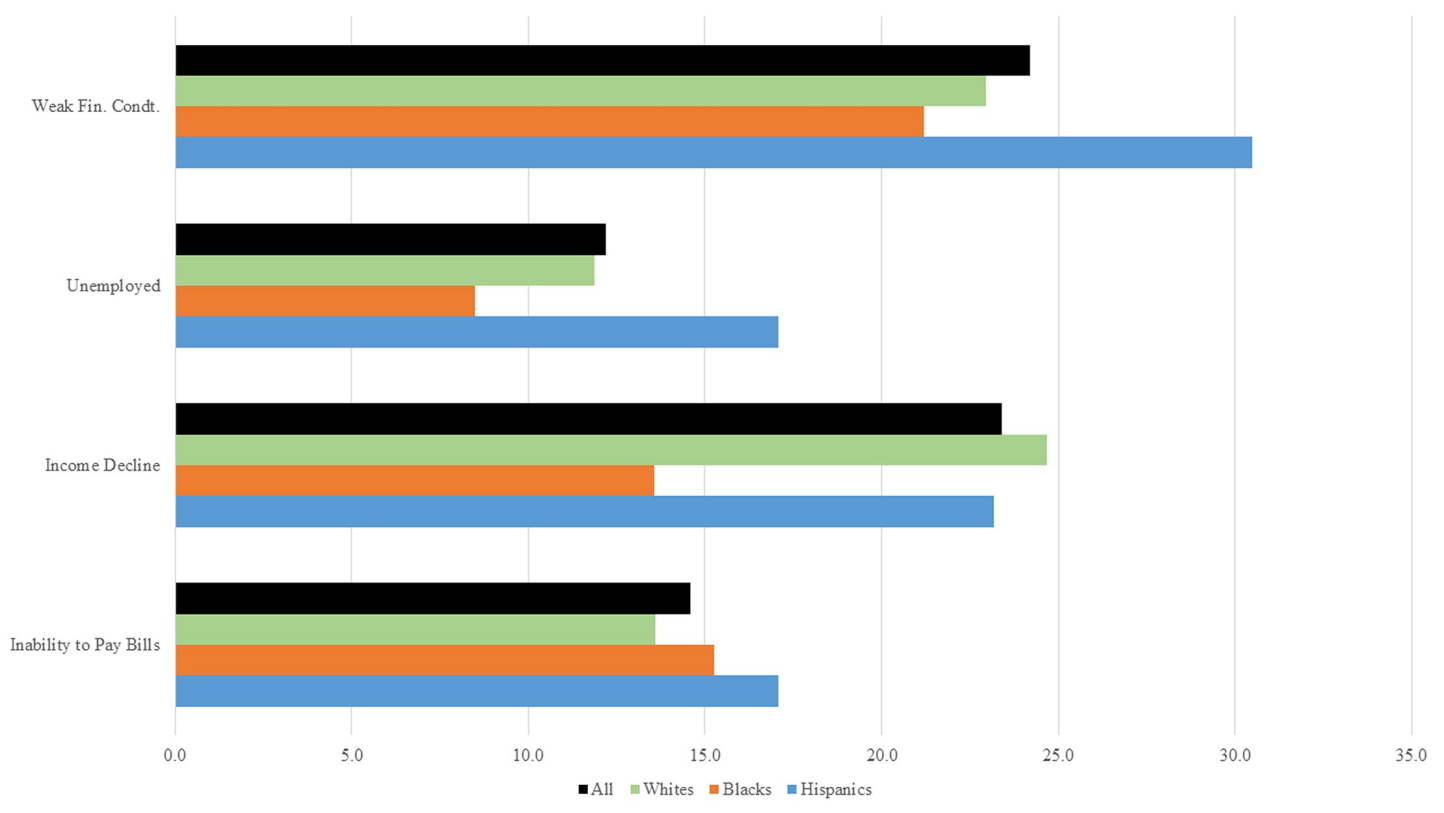
Percentage deprived in each indicator. Source: Authors calculations using SHED, April 2020, data. All percentage values are calculated using sample weights.

Fig 1 shows the percentage of individuals who were deprived in each of the four indicators. More than 20 percent of the respondents said that they were just getting by or finding it difficult to get by financially. Early on in the crisis unemployment rates soared to 12 percent; financial stress was evident from other indicators such as lower incomes compared to previous month (23 percent) and inability to pay bills in full (almost 15 percent). These values are consistent with evidence in the literature. For instance, Bartik et al. [14] used daily data on hourly workers in small businesses, and found that there was a significant decline in hours of work occurred between March 14 and March 28, 2020. Non-white workers and workers with less education were more likely to be laid off and less likely to be rehired. Between March 12 and April 12, 2020, Gupta et al. [22] found that the employment rate declined by about 1.7 percentage points for every extra 10 days that a state experienced a stay-at-home mandate.

Fig 1 also compares the percentage of individuals by race and ethnicity. Relative to other groups, a significantly higher proportion of Hispanics (30 percent) were not doing well financially. Hispanics also had a higher proportion (17 percent) among those unemployed or furloughed. Compared with Hispanics, fewer Blacks (8.5 percent) reported a job loss, yet many (21.2 percent) reported financial stress. Whites were the largest group of individuals (24.7 percent) who reported lower incomes in March 2020, compared to February 2020.

## III. Framework for multidimensional measures

Fig 1 shows a dashboard of deprivation in each indicator. Such a dashboard gives the percentage of deprived individuals in each indicator. The percentage deprived in each indicator can then be combined and expressed as some kind of an average index. For example, the Gallup-Healthways Report [23] ranks states and communities in the United States by aggregating responses in indicators such as life evaluation, emotional and physical health and so on and then calculates a composite index of wellbeing. Both the dashboard approach and the composite index fail to capture multiple deprivations experienced simultaneously by an individual. The multidimensional approach uses the individual as the unit of observation and analyzes the overlapping deprivations experienced by an individual.

This paper uses a set of indices proposed by Alkire and Foster [24]. Except for Dhongde et al. (2019) [5] who propose a different set of indices, all of the previous studies estimating multidimensional poverty in the United States, have used the Alkire and Foster [24] indices (e.g. see [4, 6, 7]) largely because of their ease of interpretation. However, note that these indices have problems associated with the methods used by them to identify the deprived and to aggregate individual deprivations (see, [25–27]).

Let *F* = {*f*_1_,…,*f*_*m*_}, *m*≥2, be a set of indicators and let *M* = {1,…*m*}. The society is denoted by *N* = {1,2,…*n*} individuals. Let *b*_*ij*_ denote the status of the *i*th individual in the *j*th indicator. If an individual chooses a response highlighted in bold in Table 1, then he/she is considered deprived in that particular indicator and *b*_*ij*_ = 1, else *b*_*ij*_ = 0. Thus, for each of the indicators, categorical data is transformed into simple binary 0–1 type of data. For instance, the question, how did your income compare to your income in the previous month, has multiple categorical responses. If an individual chooses that his/her income was somewhat lower or much lower, then he/she is considered deprived in that indicator and *b*_*ij*_ = 1. For all other responses to that question, *b*_*ij*_ = 0. Let *w*_*j*_ denote the weight assigned to the *jth* indicator and suppose each of the indicators is weighted equally, so that *w*_*j*_ = 1/*m*. Individual *i*’s weighted deprivation score is then given by $\left(\sum_{j=1}^{m}w_{j}b_{ij}\right)$. An individual is identified as multidimensional deprived, if he/she is deprived in at least *k* indicators, that is $(\sum_{j=1}^{m}w_{j}b_{ij})\geq (k/m)$. Let *Q* = {1,2,…*q*} denote a set of deprived individuals. Then the headcount ratio *HCR* = (*q*/*n*), denotes the proportion of individuals who are deprived in at least *k* indicators. Note that information on deprivation of individuals who are deprived in less than *k* indicators is not used and is one of the limitations of the identification strategy [27]. This paper overcomes the limitation by providing estimates for all possible values of *k* = {1,…,4}.

An advantage of the HCR is that it is easy to interpret since it measures the proportion of deprived individuals in the population. A drawback is that the HCR is a ratio of the deprived to the total population; it does not measure the extent of deprivation experienced by individuals. The average intensity index, A, overcomes this drawback and is calculated as $A=\frac{1}{q}\sum_{i=1}^{q}\left(\sum_{j=1}^{m}w_{j}b_{ij}\right)$. It measures the average deprivation share of the deprived individuals. It is similar to the income gap ratio of poverty. Finally, the adjusted headcount ratio, $M=\frac{1}{n}\sum_{i=1}^{q}\left(\sum_{j=1}^{m}w_{j}b_{ij}\right)$ measures the average deprivation in the entire sample and can be expressed as a product of HCR and A.

Consider the following numerical example as an illustration of the above methodology. Table 2 shows five individuals and their deprivation status in each of the four indicators. For example, individual 1 is deprived in three out of four indicators, individual 2 is not deprived in any indicator, whereas individual 3 is deprived in all indicators. Assume equal weight to each indicator. The last column in Table 2 shows the weighted sum of deprivations $\left(\sum_{j=1}^{m}w_{j}b_{ij}\right)$ for each individual.

**Table 2:** Illustrative example of multidimensional deprivation measures.

Individuals	Financial Condition	Employment Status	Income	Ability to Pay Bills	Wt. Sum of Deprivations
1	0	1	1	1	3/4
2	0	0	0	0	0/4
3	1	1	1	1	4/4
4	1	0	1	0	2/4
5	0	0	1	0	1/4

Suppose *k* = 2, then individuals with at least two deprivations, or with a weighted score of 2/4 or more, are identified as multidimensional deprived. In the example in Table 2, three out of five individuals are deprived in two or more indicators. Thus the HCR = 3/5. The average intensity index, A = (1/3) x (3/4 + 4/4 + 2/4) = 9/12, which gives the average deprivations among those identified as multidimensional deprived. Finally, the adjusted headcount ratio, M = (1/5) x (3/4 + 4/4 + 2/4) = 9/20 gives the average deprivation in the total population. It gives the actual number of deprivations as a share of the maximum possible deprivations in the population. The maximal possible deprivation in this example is 20, when all five individuals are deprived in all four indicators. As noted previously, M = HCR x A = (3/5) x (9/12) = 9/20. In the next section, these three indices are estimated using the SHED supplemental data and different values of *k*.

## IV. Estimates of multidimensional economic deprivation

Table 3 summarizes estimates of the three multidimensional indices explained in the previous section. The HCR is intuitively similar to the headcount income poverty ratio and calculates the proportion of multidimensional deprived in the population. Table 3 shows that 45.5 percent of individuals were deprived in at least one indicator. This proportion decreases as the number of indicators increases. Nearly one in four, or 24.8 percent of adults faced hardships in at least two indicators, and 10.8 percent were deprived in three or more indicators. About 3 percent adults were severely deprived, in the sense that, they were deprived in all four indicators.

**Table 3:** Multidimensional deprivation measures for the entire sample.

Deprived in at least	1 Indicator	2 Indicators	3 Indicators	All 4 Indicators
*HCR*	45.5	24.8	10.8	3.3
	(1.57) ***	(1.37) ***	(0.98) ***	(0.56) ***
*A*	0.5	0.6	0.8	1.0
	(0.01) ***	(0.02) ***	(0.03) ***	(0.07) ***
*M* = *HCR x A*	21.1	15.9	8.9	3.3
	(1.11) ***	(1.12) ***	(0.90) ***	(0.57) ***

Source: Authors calculations using formulae given in Alkire et al. [28] and SHED, April 2020, data. HCR and M values are expressed as percentages. Values are calculated for the entire sample, using sample weights. Standard errors given in brackets.

*Significant at 10%

**5%

***1%.

Recall that average intensity index, A, measures the average deprivation share among the deprived individuals. The index A always lies between zero and one. As seen in Table 3, when *k* = 1, individuals in the survey were, on average, deprived in two of the four indicators (*A* = 0.5) whereas when *k* = 3, individuals were, on average, deprived in 3.2 of the four indicators (*A* = 0.8). Finally, the adjusted head count ratio (M), combines information on the incidence (HCR) and the extent (A) of deprivation. The adjusted head count ratio gives the actual number of deprivations in the society as a share of the maximum possible deprivations. For example, Table 3 shows that when *k* = 1, the number of deprivations in the sample were 21.1 percent of the maximum possible deprivations. Table 3 also shows standard errors for all three multidimensional deprivation measures. All values are statistically significantly different from zero. Since the headcount ratio is the easiest to interpret, henceforth the results report estimates of the HCR alone, though estimates of the average intensity and the adjusted headcount ratio are available upon request.

## V. Deprivation by race and ethnicity

There is growing evidence that Covid-19 is disproportionately affecting communities comprising of racial and ethnic minority populations. In the United States, Blacks and Hispanics are overrepresented among COVID-19 cases, associated hospitalizations, and deaths. Stokes et. al. [29] analyzed demographic characteristics, underlying health conditions, symptoms, and outcomes among Covid-19 cases between January and May 2020, and found that among cases with known information, 33% of persons were Hispanics, and 22% were non-Hispanic Blacks. Hispanics and Blacks were the largest group living in hotspot counties [21]. Abedi et al. [30] found that Blacks were more vulnerable to COVID-19 than other racial and ethnic groups.

Table 4 summarizes values of the HCR by race and ethnicity. Economic deprivation was significantly greater among Blacks and Hispanics. More than 50 percent of Blacks (53.7 percent) and Hispanics (62.3 percent) experienced deprivation in at least one indicator. As seen in the Table, 37 percent Hispanics, and 29 percent Blacks were deprived in two or more indicators simultaneously, compared to about 20.5 percent Whites. The SHED survey does not collect separate data on Asians or Native Indians, and adults belonging to these groups are included in the “Other” category. In this category, 48.3 percent individuals were deprived in at least one indicator, and 28.2 percent were deprived in two or more indicators. A disproportionately high share of Hispanics (8.1 percent) were deprived in all four indicators, compared with only 3 percent in overall population. The standard errors show that most estimates were statistically significant, except for HCR estimates when k = 4.

**Table 4:** Proportion of multidimensional deprived by race and ethnicity.

	Proportion of Sample	1 Indicator	2 Indicators	3 Indicators	All 4 Indicators
White	63.2	39.0	20.5	9.1	2.6
		(1.9) ***	(1.6) ***	(1.1) ***	(0.6) ***
Black	11.8	53.7	29.1	11.2	1.7
		(4.6) ***	(4.2) ***	(2.9) ***	(1.2)
Other	7.2	48.3	28.2	7.6	1.9
		(5.9) ***	(5.3) ***	(3.1) **	(1.6)
Hispanic	16.4	62.3	37.2	18.6	8.1
		(3.8) ***	(3.8) ***	(3.0) ***	(2.1) ***
2+Races	1.4	56.7	25.1	7.9	0.0
		(13.2) ***	(11.6) **	(7.2)	--
All sample	100	45.5	24.8	10.8	3.3

Source: Authors calculations using SHED, April 2020, data. All values show the HCR in percentage terms. Values are calculated using sample weights. The groups, apart from the Hispanic, are as follows: i) White, non-Hispanic, ii) Black, non-Hispanic, iii) Other, non-Hispanic and iv) 2+ Races, non-Hispanic. Standard errors given in brackets. *Significant at 10%

**5%

***1%.

Previous studies also found higher levels of multidimensional poverty among the Hispanic compared with the rest of the population. Typically, indicators of multidimensional poverty in the United States include health insurance, high-school education, housing costs, and disabilities. Mitra and Brucker [31] found that in 2012 a greater proportion of Hispanics were deprived in three or more indicators of poverty. Dhongde and Haveman [4] found that between 2008 and 2013, about 40% of Hispanics were multidimensional poor compared with 14% in the general population. Similarly, Dhongde et al. [5] found deprivation levels to be one of the highest among Hispanics between 2008 and 2015.

## VI. Overlapping deprivations in economic indicators

**Fig 2:**
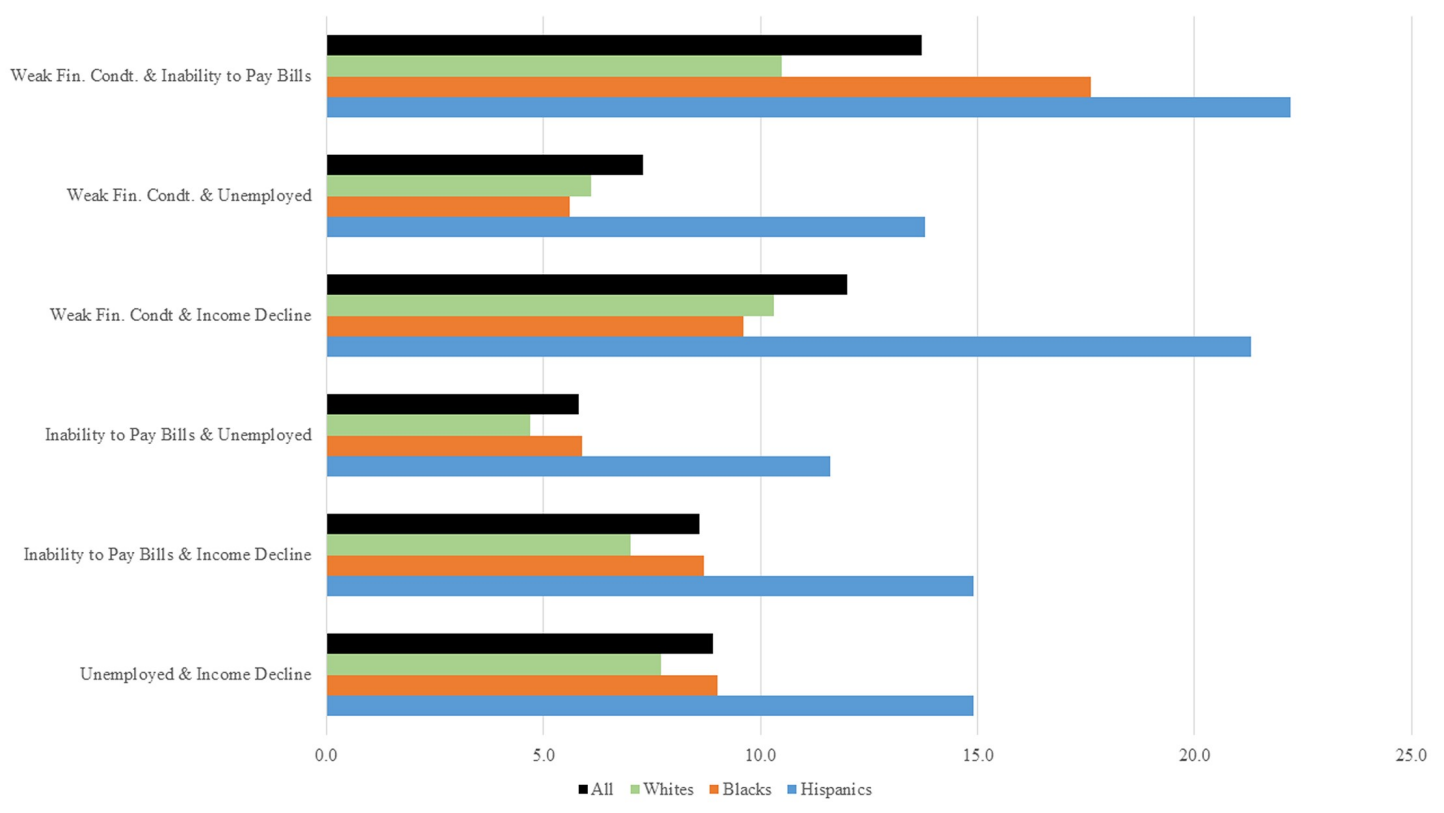
Overlapping deprivations by race/ethnicity. Source: Authors calculations using SHED, April 2020, data. All percentage values are calculated using sample weights. The groups, except the Hispanic, are as follows: i) White, non-Hispanic, ii) Black, non-Hispanic. For the sake of illustration, individuals belonging to “Other” race and “2+Races” are not included.

An important feature of the multidimensional analysis is that it identifies individuals experiencing deprivations in two or more indicators at the same time. Fig 2 shows all six pairs of indicators, in which individuals could possibly be deprived simultaneously. It also shows the proportion of individuals in the entire sample and separately among Whites, Blacks and Hispanics. In the entire sample, a large proportion of adults (13.7 percent) reported experiencing a weak financial condition and an inability to pay monthly bills in full. The correlation between deprivations in these two indicators is high (S1 Appendix). Similarly, 12 percent of the respondents had a weak financial condition and had lower income compared to previous month’s income. Thus individuals, who reported difficulty to get by financially, also reported lower incomes and an inability to pay bills. As seen in Fig 2, Hispanics stand out for high prevalence of deprivation (more than 10 percent) in each of the six combinations. Compared to 13.7 percent adults in the entire sample, 22.2 percent Hispanics and 17.6 percent Blacks reported weak financial condition and an inability to pay monthly bills in full.

## VII. Regression analysis of multiple deprivations

This section employs a regression analysis to further explore whether race/ethnicity were significant factors associated with multiple economic hardships during the pandemic. Table 5 summarizes estimated coefficients of the regression models. The estimated models include an Ordinary Least Squares (OLS) model (Seth and Tutor, 2019) [32] as well as a Poisson model, since the dependent variable is the number of deprivations experienced by an individual. The maximum likelihood estimates used in the Poisson model are similar in magnitude to the OLS estimates.

**Table 5:** Estimates from multivariate regression analysis.

Dependent Var.	OLS		Poisson	
No. of deprivations	(1)	(2)	(3)	(4)
White	-0.18	-0.07	-0.25*	-0.14
(dummy)	(0.11)	(0.11)	(0.15)	(0.15)
Black	0.07	-0.03	0.08	-0.06
(dummy)	(0.16)	(0.16)	(0.19)	(0.20)
Hispanic	0.38**	0.22	0.39**	0.17
(dummy)	(0.16)	(0.15)	(0.17)	(0.17)
Log Hh. Income		-0.25***		-0.23***
		(0.05)		(0.04)
Hh. Size		0.03		0.01
		(0.02)		(0.02)
Age		-0.01***		-0.02***
		(0.002)		(0.003)
Educ. (categories)		-0.07***		-0.09***
		(0.02)		(0.02)
Female (dummy)		0.06		0.11
		(0.06)		(0.09)
Married (dummy)		-0.10		-0.12
		(0.07)		(0.11)
Metro MSA (dummy)		-0.03		-0.10
		(0.10)		(0.13)
State Stay-at-home (dummy)		0.06		0.08
		(0.06)		(0.09)
Constant	0.08***	4.75***	-0.22	3.93***
	(0.11)	(0.51)	(0.13)	(0.50)
R-sq.(pseudo)	0.03	0.18	0.02	0.10
Total Obs.	1030	1030	1030	1030

Source: Authors calculations using SHED, April 2020, data.

* p<0.10

** p<0.05

*** p<0.01 Parenthesis shows robust standard errors. The Poisson model is statistically significant with p-value for the chi-square is equal to zero.

The basic set of results (Columns 1 and 3) confirm evidence in Section 5 that Whites had fewer deprivations and Hispanics experienced a greater number of deprivations. The coefficient from the Poisson model implies that the number of deprivations increased by 47 percent for Hispanics; the percent change is calculated as (*e*^*β*^−1)*x*100. The second set of results (Columns 2 and 4) control for household income, size and location, and individual characteristics such as age, education, gender and marital status. Interestingly, once these factors are controlled for, race or ethnicity are no longer statistically significant.

Note that SHED does not provide actual income data but imputes household income for respondents. An individual’s household income is denoted by a categorical number (between 1 and 21). In the regression analysis, each individual is assigned average income of the category to which his/her belonged. As seen in Table 5, household income had a significant negative correlation with the number of deprivations experienced by an individual. A one percent increase in household income is associated with a 20 percent decrease in the number of deprivations experienced by an individual. Table 6 shows the HCR for individuals by demographic groups. More than 65 percent of adults with incomes less than $40,000 faced economic hardship in at least one indicator, and almost 40 percent were deprived in two or more indicators. Even among those with incomes in excess of $100,000, 31.7 percent adults were deprived in at least one indicator, and 12.8 percent were deprived in two or more indicators. Chetty et al. [15] found that even high-income individuals reduced their spending sharply in mid-March 2020, particularly in areas with high rates of COVID-19 infection and in sectors that required physical interaction. Cox et al. [19] also found that all households across the income distribution cut spending from March to early April, 2020.

**Table 6:** Multidimensional deprivation by other demographic features.

	Proportion of Sample	1 dimension	2 dimensions	3 dimensions	All 4 dimensions
Gender					
Male	48.4	43.0	24.5	9.3	2.8
Female	51.6	47.9	25.2	12.1	3.8
Age					
18–29	20.9	61.4	36.0	16.9	6.8
30–44	25.1	50.0	31.3	12.2	4.0
45–59	24.7	48.0	26.7	12.9	2.3
60+	29.3	28.2	9.8	3.3	1.0
Education					
Less than high school	10.6	66.4	40.3	14.9	3.9
High school degree	28.3	49.6	29.5	12.0	3.2
Some college	27.8	56.7	31.6	15.0	5.5
Bachelor's degree	33.3	25.9	10.3	4.8	1.3
Marital Status					
Married	53.6	37.0	17.6	6.7	1.3
Living w/Partner	5.8	60.4	29.0	20.0	14.6
All others	40.6	54.5	33.8	14.8	4.3
Household Size					
1 to 2	56.2	38.6	19.8	8.5	2.5
3 to 5	38.2	52.1	29.6	13.3	4.4
6+	5.6	69.9	43.0	16.4	4.2
Household Income					
Less than $40,000	24.9	65.6	39.4	19.4	5.9
$40,000-$99,999	36.9	46.2	27.4	11.4	3.9
$100,000+	38.2	31.7	12.8	4.5	1.0
MSA					
Non-Metro	12.6	46.8	26.3	11.5	5.6
Metro	87.4	45.3	24.6	10.6	3.0
All sample	100	45.5	24.8	10.8	3.3

Source: Authors calculations using SHED, April 2020, data. All values show the HCR in percentage terms. Values are calculated using sample weights. Under Marital Status, “All others” include widowed, divorced, separated, and never married. Standard errors not included for brevity.

In addition to income, the estimated regression coefficients shows that individual’s, age and education had a significant negative correlation with deprivation. Table 6 shows that the depletion of economic wellbeing among young adults (18–29 year olds) was significantly higher compared to other age groups. Among young adults, 61.4 percent were deprived in at least one indicator (compared with 45.5 percent in the overall sample), and 36 percent were deprived in at least two indicators (compared with 24.8 percent in the overall sample). Adults with less education suffered more economic hardships. Almost 40 percent of high school dropouts and 31 percent of those without a college degree faced economic hardship in at least two of the four indicators.

In Table 5, the dummy variable on MSA (metropolitan statistical area) indicates whether the individual resided in a metro or non-metro MSA. Additionally, a dummy variable was created for households located in states where stay-at-home orders were issued before April 1, 2020, that is before the survey was conducted. There were 31 states with such orders according to data compiled from the New York Times [9]; states with partial orders issued only for some cities were not included. Table 5 shows that neither the MSA type nor the dummy variable on states with stay-at-home orders was a significant predictor of the number of deprivations experienced by individuals.

## VIII. Conclusions

Worldwide, as the number of Covid-19 cases and resulting deaths continue to rise, there is a growing concern not only about global health but also about a looming economic crisis. Most of the economic impact of the virus results from aversion behavior, the actions people take to avoid catching the virus [33]. Importantly, as the number of Covid-19 cases rise dramatically, especially among developing countries including India, Russia, Brazil, Peru, Chile, and Iran, the pandemic is also having a detrimental impact on the global poor. The International Labor Organization (ILO) predicted that in 2020 between 9 and 35 million additional people will be added to the working poor in developing countries [34]. Even as poor countries continue to struggle to manage the crisis, it is also evident that the rich and more developed countries have not been spared by the coronavirus pandemic and the resulting economic shock. This paper measured the rapid depletion of economic wellbeing in the United States in the early stages of the pandemic.

So far, few surveys have been conducted during the pandemic. The paper used one such publicly available datasets in the United States. The timing of the survey is important since it was conducted in the midst of the coronavirus pandemic when stay-at-home orders were issued across the country. The survey was conducted after the passage of the CARES Act, but before most benefits of the Act were received. A limitation of the analysis is that the supplemental SHED survey had a small set of questions related to economic hardship. For instance, there were no questions on food insecurity or housing evictions; two of the most important fallouts from the resulting economic shock.

The analysis used four indicators of economic hardship, namely overall financial condition, loss of employment, reduction in income compared to that in the previous month and inability to pay monthly bills. Instead of simply noting the proportion of individuals deprived in each of the four indicators, the paper employed a multidimensional analysis to measure the overlapping economic deprivations experienced individuals during the early stages of the pandemic in the United States.

Measures of multidimensional deprivation showed that almost 25 percent of the respondents faced hardships in at least two of the four indicators. More than 13 percent individuals reported their inability to pay monthly bills and struggled to make ends meet financially. One in four respondents had lower income compared to income from previous month. The economic setback affected Hispanics in a more profound way than any other racial/ethnic group. More than 37 percent Hispanics reported hardship in two or more indicators and 8 percent reported hardship in all four indicators. Overall, higher proportion of young adults and those without a college degree suffered multiple economic hardships. Results confirm that economic disparities deepened along racial/ethnic lines. A recommended policy response is to prioritize economic aid to vulnerable populations; the racial and ethnic minorities, young adults with low incomes and poor educational backgrounds. A step forward, in terms of research, will be to use additional data as it becomes available, to analyze whether deprivation deepened during the later stages of the pandemic and whether policies under the CARES Act were effective in reducing economic hardships among the most vulnerable populations.

## Supporting information

S1 Appendix Correlation between each indicator. (DOCX)
