# Supplementary material for: Multidimensional economic deprivation during the coronavirus pandemic: Early evidence from the United States
Source: PLoS One. 2020 Dec 16;15(12):e0244130. doi: 10.1371/journal.pone.0244130 (PMC7744060; doi:10.1371/journal.pone.0244130)
Supplement: S1 Appendix — (DOCX) [file pone.0244130.s001.docx]

# **S1 Appendix: Correlation between Each Indicator**

|  | Weak Fin. Condition | Inability to Pay Bills | Unemployed | Income Decline |
| --- | --- | --- | --- | --- |
| Weak Fin. Condition | 1 |  |  |  |
| Inability to Pay Bills | 0.78 | 1 |  |  |
| Unemployed | 0.44 | 0.47 | 1 |  |
| Income Decline | 0.47 | 0.46 | 0.64 | 1 |

Source: Authors calculations using SHED, April 2020, data. Table gives values of tetra choric correlation coefficients, which are used when variables are binary, 0-1 in our case. All tetra choric correlation coefficients are significant at 5%.
